# A three-dimensional finite element analysis of mechanical function for 4 removable partial denture designs with 3 framework materials: CoCr, Ti-6Al-4V alloy and PEEK

**DOI:** 10.1038/s41598-019-50363-1

**Published:** 2019-09-27

**Authors:** Xin Chen, Bochun Mao, Zhuoli Zhu, Jiayi Yu, Yuqing Lu, Qianqian Zhang, Li Yue, Haiyang Yu

**Affiliations:** 1 Department of Prothesis, West China Hospital of Stomatology, Sichuan University, Chengdu, 610000 China; 2 the State Key Laboratory of Oral Disease, West China College of Stomatology, Sichuan University, Sichuan, 610041 China

**Keywords:** Oral anatomy, Computational methods

## Abstract

Polyetheretherketone (PEEK) is a new material used for the frameworks of removable partial dentures (RPD). The questions whether the PEEK framework has similar stress distribution on oral tissue and displacement under masticatory forces as titanium alloy (Ti-6Al-4V) or cobalt-chromium alloy (CoCr) remain unclear and worth exploring. A patient’s intraoral data were obtained via CBCT and master model scan. Four RPDs were designed by 3Shape dental system, and the models were processed by three-dimensional finite element analysis. Among three materials tested, PEEK has the lowest maximum von Mises stress (VMS) on periodontal ligament (PDL), the greatest maximum VMS on mucosa, the maximum displacement on free-end of framework, and the lowest maximum VMS on framework. Results suggested that PEEK framework has a good protective effect on PDL, suggesting applications for patients with poor periodontal conditions. However, the maximum displacement of the free-end under masticatory force is not conducive for denture stability, along with large stress on the mucosa indicate that PEEK is unsuitable for patients with more loss of posterior teeth with free-end edentulism.

## Introduction

Removable partial dentures (RPDs) have wide indications and flexible designs compared to fixed partial dentures or implant restorations^1,2^, and they are frequently used to restore dentition defects during the clinical work of prosthesis. Conventional RPD framework materials could be roughly categorized into metals and non-metals. Cobalt-chromium (CoCr) alloy is one of the most common metal RPD framework material. However, several severe problems of CoCr alloy, such as aesthetic problems and metallic in taste, were pointed out in several researches^3–5^, and these problems are likely applicable to other metal materials. Nonmetal denture framework materials includes a large body of organic polymers accompanied by a wide spectrum of physical and chemical properties. The dental profession always thrives for better materials which can fulfill the pitfalls of the existing materials. The anti-allergic property, polishability, low plaque affinity, and wear resistance of the nonmetal materials can be improved by using polyetheretherketone (PEEK), which is a semi-crystalline organic polymer with stable chemical properties, high biocompatibility, high temperature resistance, and easy mechanical processing properties^3,6^. All these features of PEEK could be considerably attractive for clinicians who seek improvements over conventional dental materials^7^. Recently, PEEK has been used for the implant supported bridge and claps of RPDs^7–10^, and only one published study focused on the design of integrated RPD with the material^11^. Because of its color and other physical properties, PEEK permits RPD fabrication with esthetic clasps and occlusal rests with better occlusal stability^11^.

Although the supreme physical and chemical properties of PEEK were clearly demonstrated in the literature, to serve the practical purpose of designing better dental structure, compatibility between the material and specific RPD frameworks is always a non-trivial topic that requires careful discussion. Since different design schemes with different materials have different effects on the soft and hard tissues of the patients, improper design scheme can damage patient’s periodontal and mucosal health. For the RPDs of patients with deletion of Kennedy Class I, such damage can be more significant. For these patients, the free-end abutment is exerted with stress on the cantilever beam, where a certain sinking occurs during mastication. The instability of the framework reduces the masticatory efficiency of the patient. In addition, the difference between the stress response and the elastic deformation caused by periodontal ligament (PDL) and mucosa is likely to cause excessive distal and medium-direction torque, which may cause bone restoration of the abutment alveolar ridge, leading to loosening or even detachment of the abutment^12–14^.

There is a variety of existing methods to investigate the mechanical behaviors in dentistry such as photoelasticity measurement, strain gauge-based measurements, optic measurement, and computational approaches^15^. Computational methods, if chosen carefully, could provide reliable data with high efficiency. In this study, Finite Element Analysis (FEA) is found to be particularly suitable for our purposes. FEA is a numerical method uses variational methods from the calculus of variations to approximate a solution by minimizing an associated error function after subdividing a large system into parts and then assembling them into a larger system of equations that models the entire problem^16^. FEA has been widely used to predict the biomechanical behavior of various types of prosthetic bodies in oral environment^17^, such as dental implants^18^, removable and fixed prosthesis^19^, implant-assisted RPD^20^ and bone metabolism^21^. This method is quite useful for exploring mechanical behaviors of tissues that can hardly be investigated *in vivo*.

However, the conventional way of reconstructing mucosa for FEA is not accurate enough for analyzing the stress distribution or displacement of mucosa since the morphology or the thickness of mucosa is excessively simplified. One conventional way of reconstructing mucosa requires expanding the mandible bone surface at an even thickness (~2 mm). But the thickness of mucosa in patients’ mouth varies much among different areas of oral cavity^22^, using one single parameter to represent the thickness could be at best a rough approximation. Another common method of reconstructing mucosa exploits CBCT or CT data to reconstruct mucosa together with other hard tissues. However, it is mentioned in both basic principles of using CBCT of Europe and America^23,24^ that CBCT should not be used to evaluate the soft tissues due to the poor quality compared with CT or MRI. Therefore, the common two methods cannot fulfill the requirement for FEA that aimed to study the stress distribution or displacement of oral mucosa. This research revealed a new way of reconstructing mucosa more accurately with accurate thickness and morphology of mucosa reserved by involving both optical scan data and CBCT data.

Hence, this article aimed to explore the influence of PEEK as RPD framework material on the patient’s oral tissues through FEA and whether the key design points of the framework with PEEK are similar to those of cobalt chromium (CoCr) and titanium (Ti-6Al-4V) alloys with a more accurate simulation method of oral mucosa.

## Results

**Figure 1:**
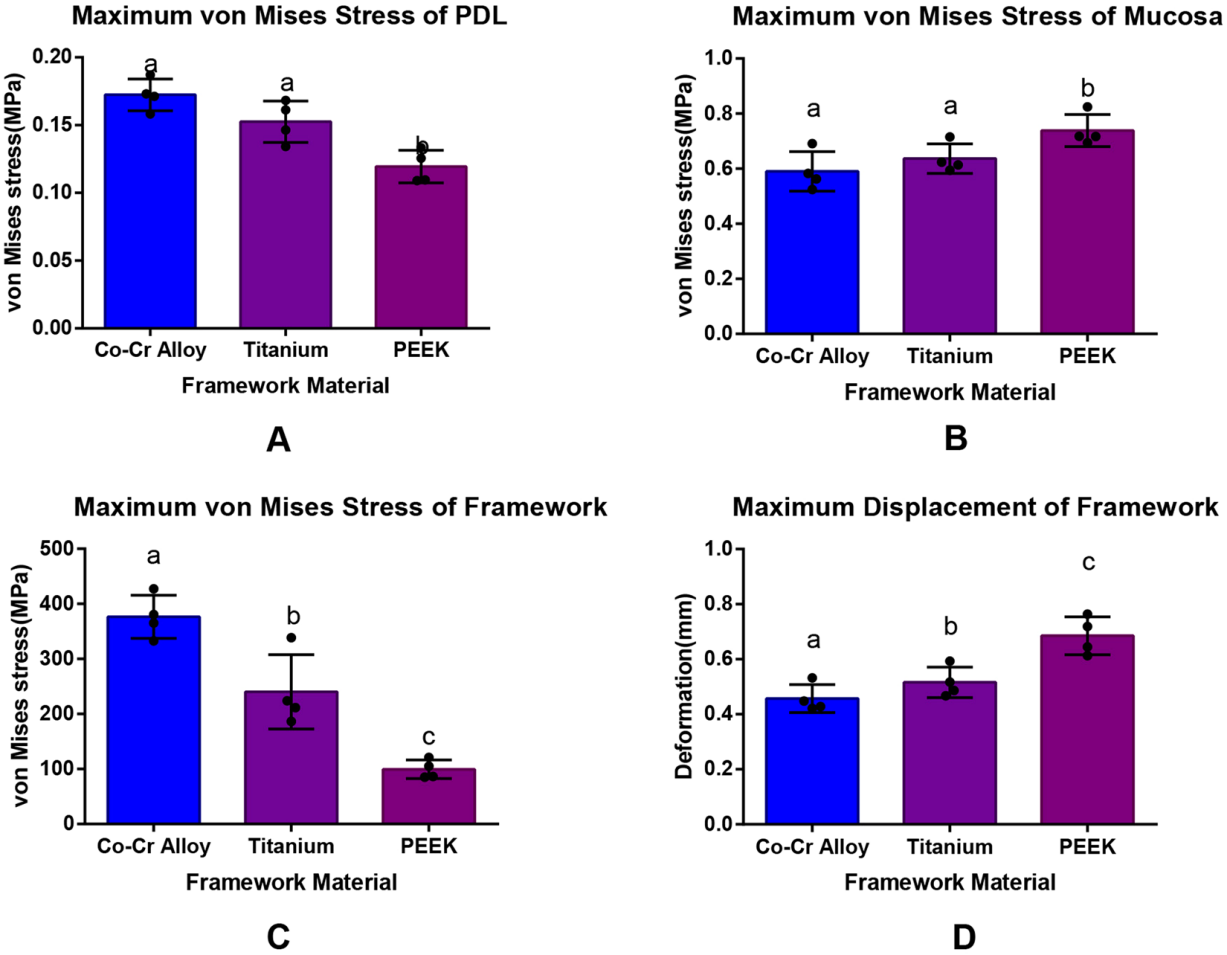
Max von Mises stress values and displacement of models under vertical loading.

Compared with CoCr and Ti-6Al-4V alloy, PEEK has the lowest maximum von Mises stress on the PDL, the greatest maximum von Mises stress on the mucosa, the largest displacement on the free-end and the lowest maximum von Mises stress on the framework with significant differences compared with those values of the other two materials (*p* < 0.05, SNK q test, Fig. 1).

**Figure 2:**
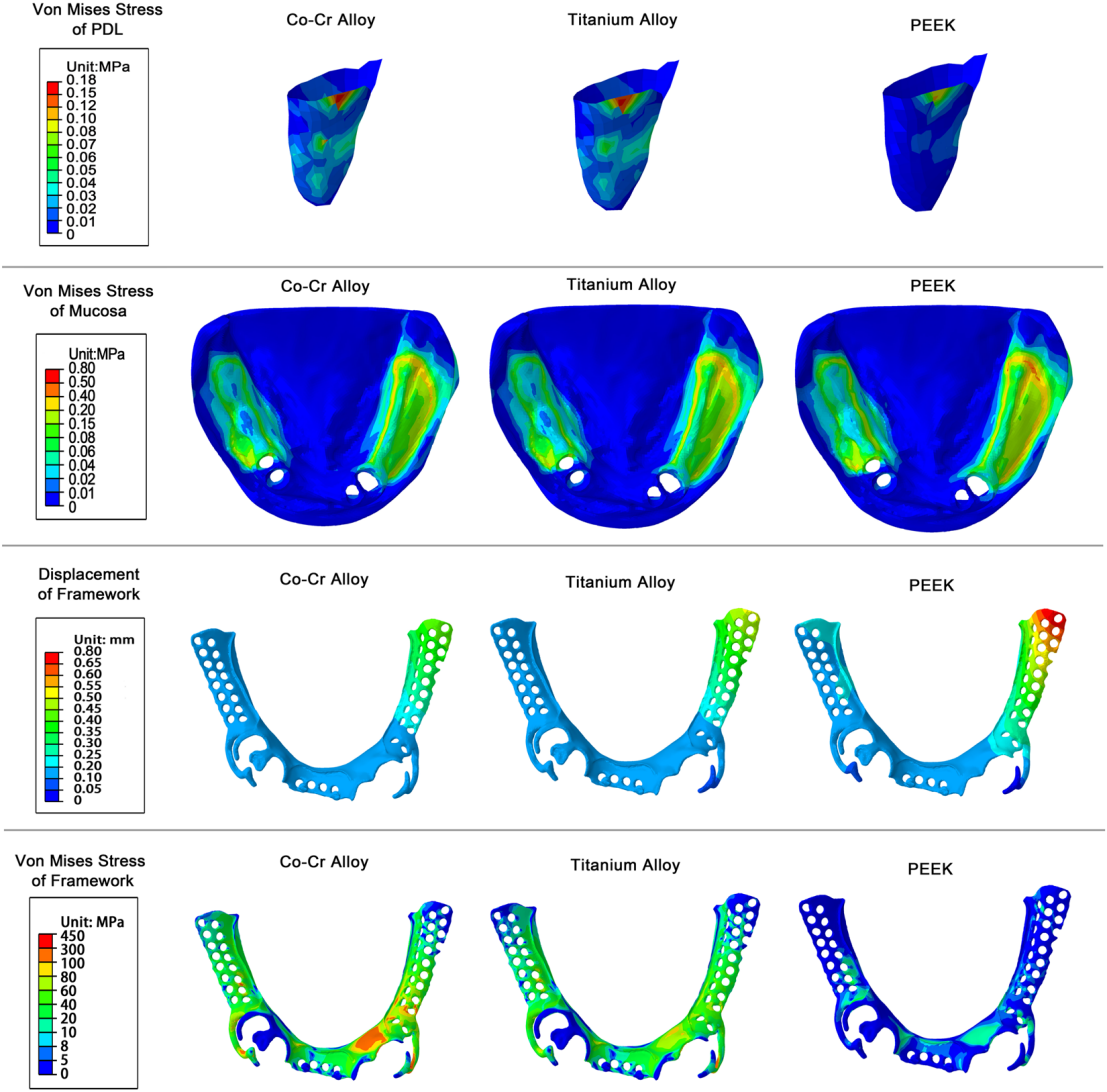
von Mises stress of PDL, mucosa, framework and displacement of framework under vertical loading.

The maximum von Mises stress of the PDL of CoCr alloy, Ti alloy, PEEK groups were 0.17 ± 0.01 MPa, 0.15 ± 0.02 MPa, 0.12 ± 0.01 MPa respectively (mean ± SD), indicating significant difference between PEEK with the other two materials (*p* < 0.05, SNK q test). The maximum stress of the PDL of PEEK group was only 77.8% of the Ti alloy and 69.2% of the CoCr alloy, indicating that PEEK was more conducive in the protection of PDL of the abutment. All the stress concentration points of the PDL were located at the distal cervix of the left lateral incisor of the lower jaw (Fig. 2).

Meanwhile, the maximum mucosa stress of the frameworks made with PEEK was 0.74 ± 0.06 MPa (mean ± SD), which was significantly larger than the other two materials (*p* < 0.05, SNK q test). No statistical difference was found between the other two materials (Ti alloy: 0.64 ± 0.05 MPa (mean ± SD); CoCr alloy: 0.59 ± 0.07 MPa (mean ± SD)). The stress distribution positions of the mucosa of the three materials were similar, with the maximum displacement points occurring at the retromolar pad area of the left side of the mandible.

As for framework maximum displacement, the free-end displacement of the framework made by PEEK was the largest with the value of 0.69 ± 0.07 mm (mean ± SD), which was significantly larger than Ti alloy (0.52 ± 0.06 mm) and CoCr alloy (0.46 ± 0.05 mm) (mean ± SD, *p* < 0.05).

The maximum von Mises stresses of the CoCr alloy, Ti alloy and PEEK framework were 375.48 ± 39.31 MPa, 240.17 ± 67.52 MPa and 99.54 ± 16.98 MPa (mean ± SD) respectively. There were significant differences among the three materials (*p* < 0.05, SNK q test). The maximum stress of PEEK was 41.4% that of the Ti alloy and 26.5% that of the CoCr alloy, suggesting that the PEEK significantly reduced the internal stress of the framework.

## Discussion

RPD is one of the most commonly used prostheses to restore dentition defects, and is used extensively in several indications including deletion of Kennedy Class I. As the functional basis of the denture, the integrated framework offers connection, retention and stability functions. The abutment serves as an important source of retention force when attached to various types of retainers. The mucosa, alveolar bone and other tissues provide support for the denture, and it is vital for the denture base to fully cover the supporting tissues in order to disperse the masticatory force. Clearly, poor condition of the abutment and undesired PDL changes under improper stress would directly impact the restorative effect of the RPD. Also if the mucosa and other relevant tissues are unable distribute stress evenly, the tension would lead to local tenderness or excessive absorption of the alveolar bone, affecting the use of denture^13,14^. Therefore, a proper framework design should take into consideration not only the retention and stability of the denture, but also the stress on the abutment and relevant tissues.

The flexibility of the framework design attributes to varied kinds of transmission and distribution of masticatory force, which results in different distributions of stress in oral tissues. Based on the characteristics of bilateral dissociation deletion of Kennedy Class I, we designed four different frameworks for the patient enrolled by taking into account the design conceptions of different dentists and technicians as much as possible. For framework A, an RPT (Rest-Plate-T bar) clasp was set in the canine of left mandibular region and an RPI (Rest-Plate-I bar) clasp was set in the second premolar of right mandibular region to act as stress-broker and reduce both distal movement of the abutment and exposure of clasps. For framework B, an RPT clasp was used as in framework A, and an arrow clasp, which also served as stress-broker, was set between the premolars of the right mandibular region. In framework C, an RPT clasp was set as above reason, while an RPL (Rest-Plate-L bar) clasp and a back-action clasp, were used in the right mandibular region to provide higher retention force and stress-broken effect. In framework D, a combined clasp was set between the premolars of right mandibular region to reduce the distal forces on 45 and to reduce the exposure of clasp on 44. In addition, an Aker clasp was set in the canine of left mandibular region to enforce retention. The above 4 framework design are often used in clinical practice.

Stress of PDL must be taken into account in order to foresee the root resorption and bone remodeling processes, which seems to be generated by PDL stresses^16,25,26^. Furthermore, PDL stresses correlate with the bone necrosis process strongly. It has been established that this process would only begin if the stress along the fibres of PDL exceeds the pressure value inside the blood capillary vessels in the alveolar region^27^. In this experiment, the max stress of the PDL of the four remaining teeth appeared at the PDL of the mandibular left lateral incisor cervix. This was because the tooth was presented on the side of the one free-end edentulous dentition with a longer distance between this tooth and the distal end of the framework. Besides, lingual plates were adapted in these four different designs. The lingual plate was in contact with the lingual side of the tooth, which can prevent the framework from sinking. The force is transmitted to the tooth and then to the PDL while chewing. Taking both effects into consideration, the maximum stress concentration point of the PDL appears at the very place, which was consistent with the previous researches^28^. Under same loading conditions, PEEK framework demonstrated the best protection function on the PDL, which may be particularly suitable for patients with poor periodontal conditions.

In our patient’s case, there were more missing teeth on the left free-end side of the lower jaw than the right side. In the language of mechanics, the stress arm of dislocation force on the left side of the lower jaw is longer. However, in each of the frameworks there was only one direct retainer on the left side, which was located on the left canine of the lower jaw. The direct retainer on the right side of the mandible is located on the two premolars, indicating that the left retaining force is smaller. Under similar torque conditions, the dislodging force on the left side of the mandible was larger. Therefore, regardless of the framework material and design, the stress of the framework was larger on the left side. By comparing the framework A-C (T bar) with the clasp on the left side of the framework D (Aker clasp), it was found that the stress concentration point of the framework was often located at the right-angle turn between the clasp and minor connector (Fig. 2), which was consistent with previous research^29^. The stress of PEEK framework was smaller and more evenly distributed compared to CoCr and Ti-6Al-4V alloys with 73.5% smaller maximum stress than that of the CoCr alloy. However, whether PEEK has a better long-term behavior against fatigue fracture is still unclear, which is not only determined by a smaller stress but also by the fatigue resistance of the material

However, the displacement of the free end of the PEEK framework was larger, which was a disadvantage regarding the stability of the denture and may cause failure of the wrapped PMMA denture base. A previous case report^30^ which investigate a Kennedy Class I case also found that PEEK framework may cause a lower retentive force compared with metallic frameworks due to the low modulus of elasticity of the material^3^. The larger displacement may also reduce the chewing efficiency of the patient.

In papers, stresses beneath the denture caused by masticatory forces measures both in the lab or by simulation were about 80–300 Kpa^31–35^. Taking into account the influence of the differences in foundation and occlusal loads on stress values, there is a convergence between the results of this paper and previous studies. As shown in the result, PEEK frameworks exhibit a significant larger stress on mucosa compared to other two metallic materials, which may lead to increased absorption of the alveolar bone^36^. Previous studies pointed out the pain threshold of mucosa is at the level of approximately 0.63 MPa^37,38^, which called for caution to be taken while using PEEK frameworks with patients with free end teeth loss.

This study has put forwarded a new method to establish the three-dimensional finite element model of mucosa for analyzing the mechanical behaviors of denture in the oral cavity. One common conventional method^29,39,40^ is to use only CBCT scan data to reconstruct the mandibular bone and simulate the mucosa as a 2 mm thickness expansion of the bone. This method retained the details of bone and dentition at a higher level; however, the single 2 mm thickness parameter offers researchers too less information about the oral mucosa, which meant that this kind of model was not suitable for analyzing the stress of mucosa, especially for cases with mucosal support dentures. On the contrary, another conventional method uses only the master model scanned data without using the CBCT scan to build the mucosa model. Within this method, the dental technicians grind down 2 mm plaster from the master model to simulate the bone. Although it can reveal the features of oral mucosa, the consistent 2 mm thickness still could not provide the exact thickness of oral mucosa and accurate morphology of the bone. In contrast to the above two methods, this article provided a solution for revealing the detailed features of both mucosa and jaw bone. By fitting the CBCT data and the master model scanned data together by aligning the remaining teeth to gain mucosa model, this method could be much more suitable for analyzing the mechanical behaviors of mucosal support denture with three-dimensional FEA.

However, there are also several limitations with this study. In this study we only enrolled one patient with one type of edentulous arches (Kennedy Class I). More types of edentulous arches with more cases are needed to give a much stronger point. What is more, our patient has a prolonged history of tooth loss, which caused severe alveolar bone resorption that led to a reduction of PDL area. The smaller area of PDL may cause more significant stress concentration in PDL and displacement of the teeth. Moreover, the anatomical shape of the roots was not satisfyingly proper, which may be explained by the not so significant difference of contrast between the root and cancellous bone in the CBCT data.

## Conclusions

Results of this study suggest that the framework made of PEEK has better protection function for PDL with more even dispersion of masticatory force compared with conventional metallic materials, which is suitable for patients with poor periodontal conditions. However, the stress on the mucosa under the denture and the displacement of the free end of the PEEK framework is larger, which is a disadvantage for the stability of the denture and may cause pain in mucosa. Caution should be taken while using PEEK framework in patients with free end teeth loss.

## Methods

**Figure 3:**
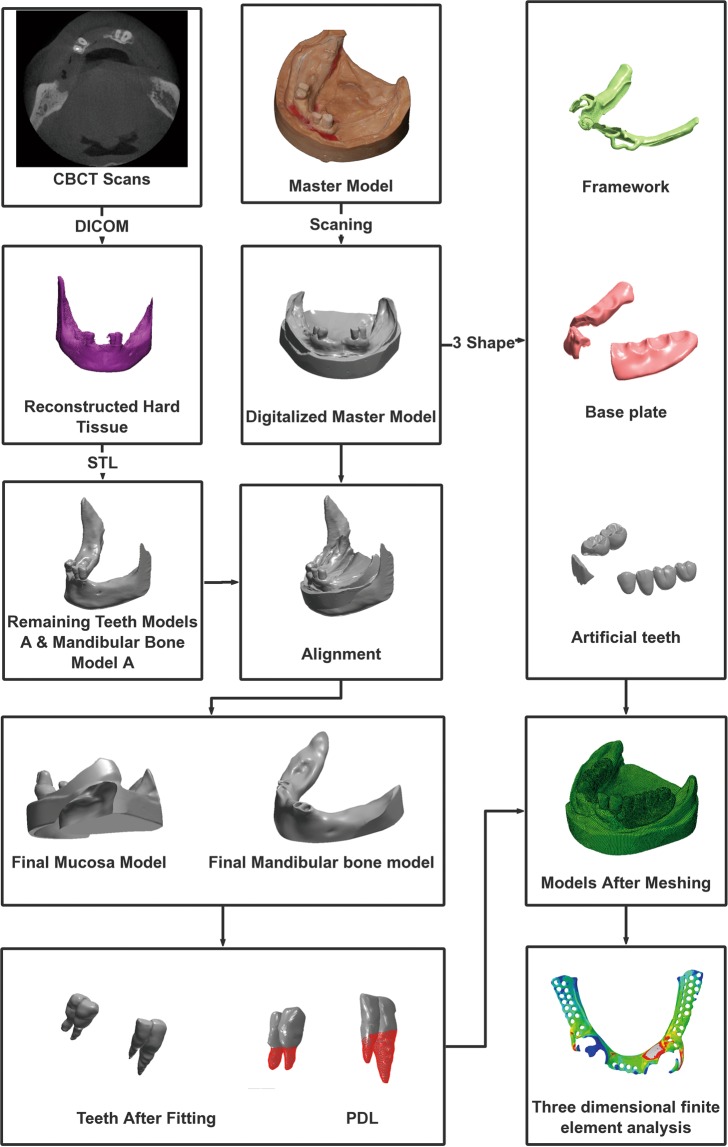
The flow chart of modeling.

Experimental procedures were approved by the West China Hospital of Stomatology, Sichuan University (reference number WCHSIRB-D-2018-105). All methods were performed in accordance with the relevant guidelines and regulations in the informed consent. And the informed consent was gotten to publish identifying information including CBCT and oral master model. A 3-dimensional FEA solid model of lower jaw was constructed using clinical CBCT data and master model scanned data from a 53-year-old Chinese patient with bilateral dissociation deletion of Kennedy Class I. Oral examination showed a severely absorbed mandibular bone with 34–37, 31–43, 46, and 47 teeth missing (FDI standard), and no other abnormality was discovered among the remaining teeth. The modeling steps are shown in Fig. 3.

The mandibular bone was scanned with CBCT (3D Accuitomo scanner, Morita, Kyoto, Japan) at a 0.25-mm-slice thickness and 1-mm scan increment in 401 slice images in DICOM format. The images were imported into the Mimics (Mimics 17.0, Materialise NV) to obtain the mandibular bone model and the remaining teeth models. The output data were then imported to Geomagic Studio (Geomagic Studio12.0, Geomagic Co, USA) for surface reconstruction. The remaining teeth models A (RTMA) and mandibular bone model A (MBMA) were obtained for further use.

The mandibular impression was made by using alginate impression materials, and the master model was made with plaster. The features of oral mucosa were obtained by scanning the master model with a desk scanner (3shape D2000, Denmark), and the obtained data were imported to Geomagic Studio for surface reconstruction. Digitalized master model (DMM) was obtained for further use.

**Figure 4:**
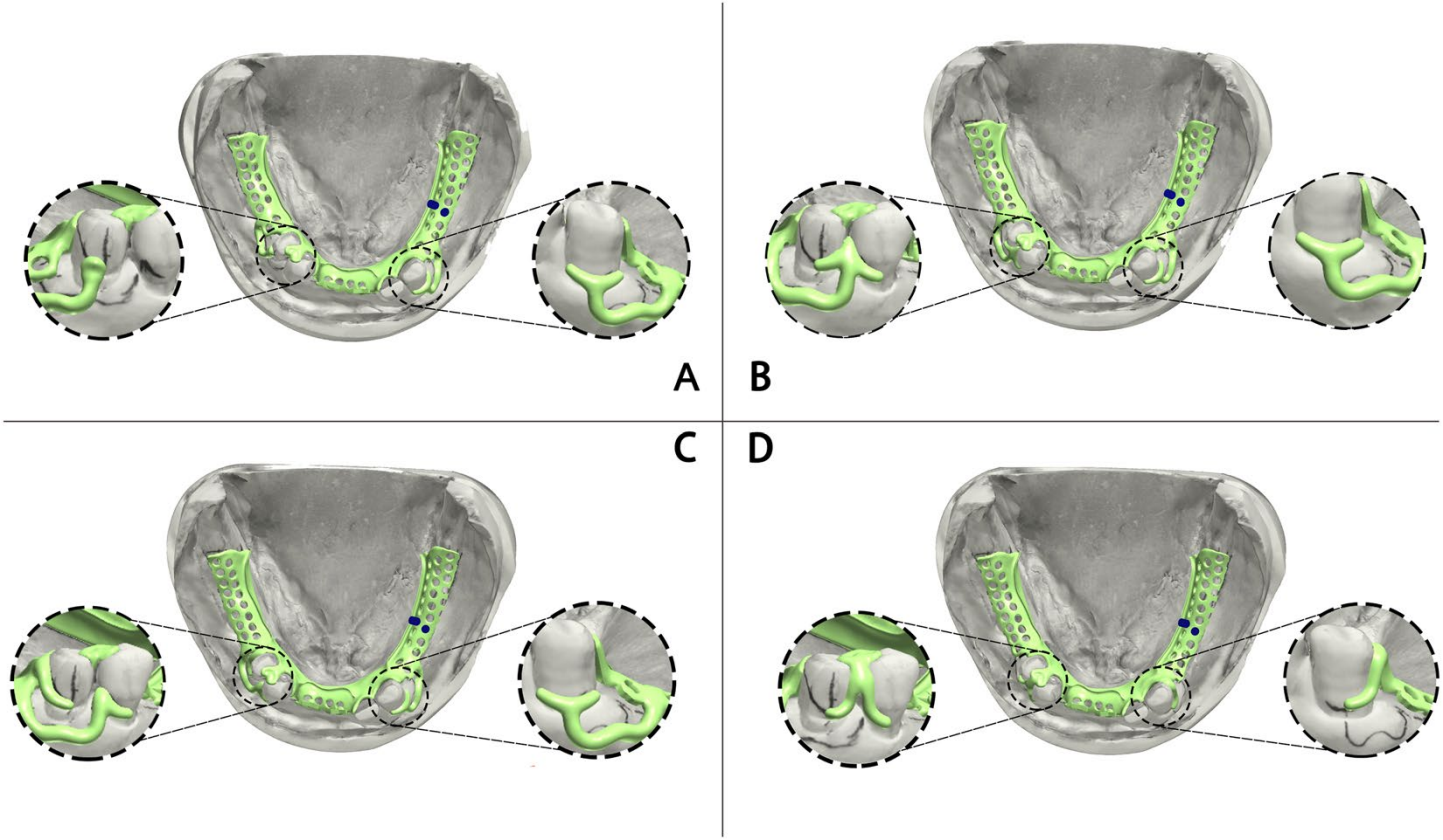
4 Different designs of frameworks.

RTMA, MBMA and DMM were aligned in Geomagic Studio by matching three matching points on the remaining teeth surfaces of RTMA and DMM. Boolean operation was first used to remove the exposed part of bone of MBMA to obtain the final mandibular bone model (FMBM). Boolean operation was then used to remove the remaining crowns on the DMM by subtracting it with RTMA, and the final mucosal model (FMM) was obtained. The mucosa thickness was defined with the vertical distance between the mandibular bone surface and the mucosal surface. The 3D oral model obtained with this technique can provide a more accurate geometrical morphology and thickness of mucosa, which is crucial for RPD simulation. The PDL was simulated by adding a 0.2 mm thick shell to the interface area between bone and tooth models, and then the volume shell is subtracted from the bone in order to define the PDL volume as previous studies proposed^41,42^. Following the design specifications for RPDs, 4 kinds of RPDs were designed by an experienced prosthodontist as well as technician together by using 3shape Dental System software (Dental system 2017, Denmark). Figure 4 demonstrated the 4 different designs of frameworks, and the designs are as follows:

Framework A: an RPT (Rest-Plate-T bar) clasp set in the canine of left mandibular region; an RPI (Rest-Plate-I bar) clasp set in the second premolar of right mandibular region.

Framework B: an RPT (Rest-Plate-T bar) clasp set in the canine of left mandibular region; an arrow clasp between the premolars of the right mandibular region.

Framework C: an RPT (Rest-Plate-T bar) clasp set in the canine of left mandibular region; an RPL (Rest-Plate-L bar) clasp set in the first premolar of right mandibular region; a back-action clasp in the second premolar of right mandibular region.

Framework D: an Aker clasp in the canine of left mandibular region; a combined clasp between the premolars of right mandibular region.

**Figure 5:**
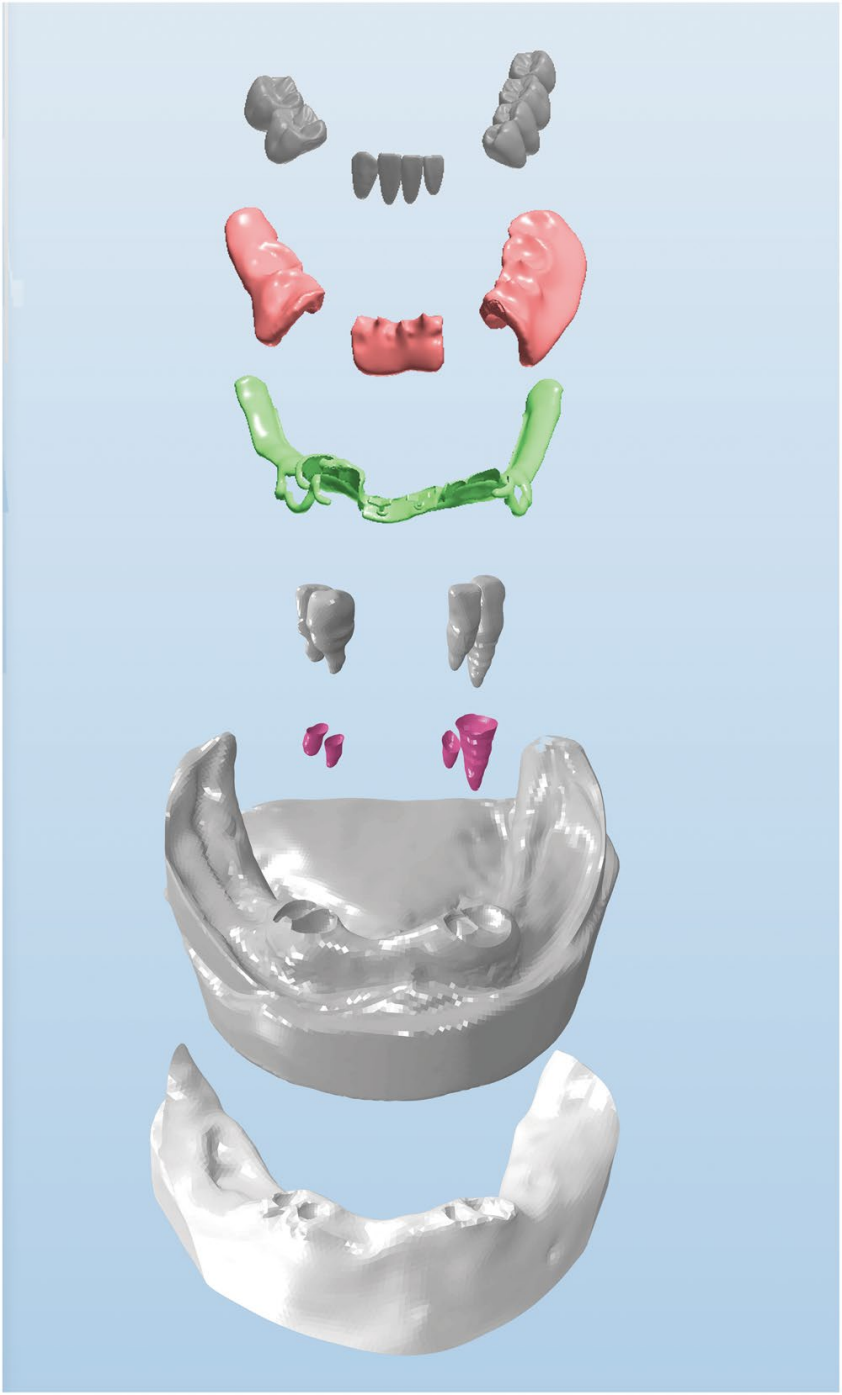
Final components of the model.

**Figure 6:**
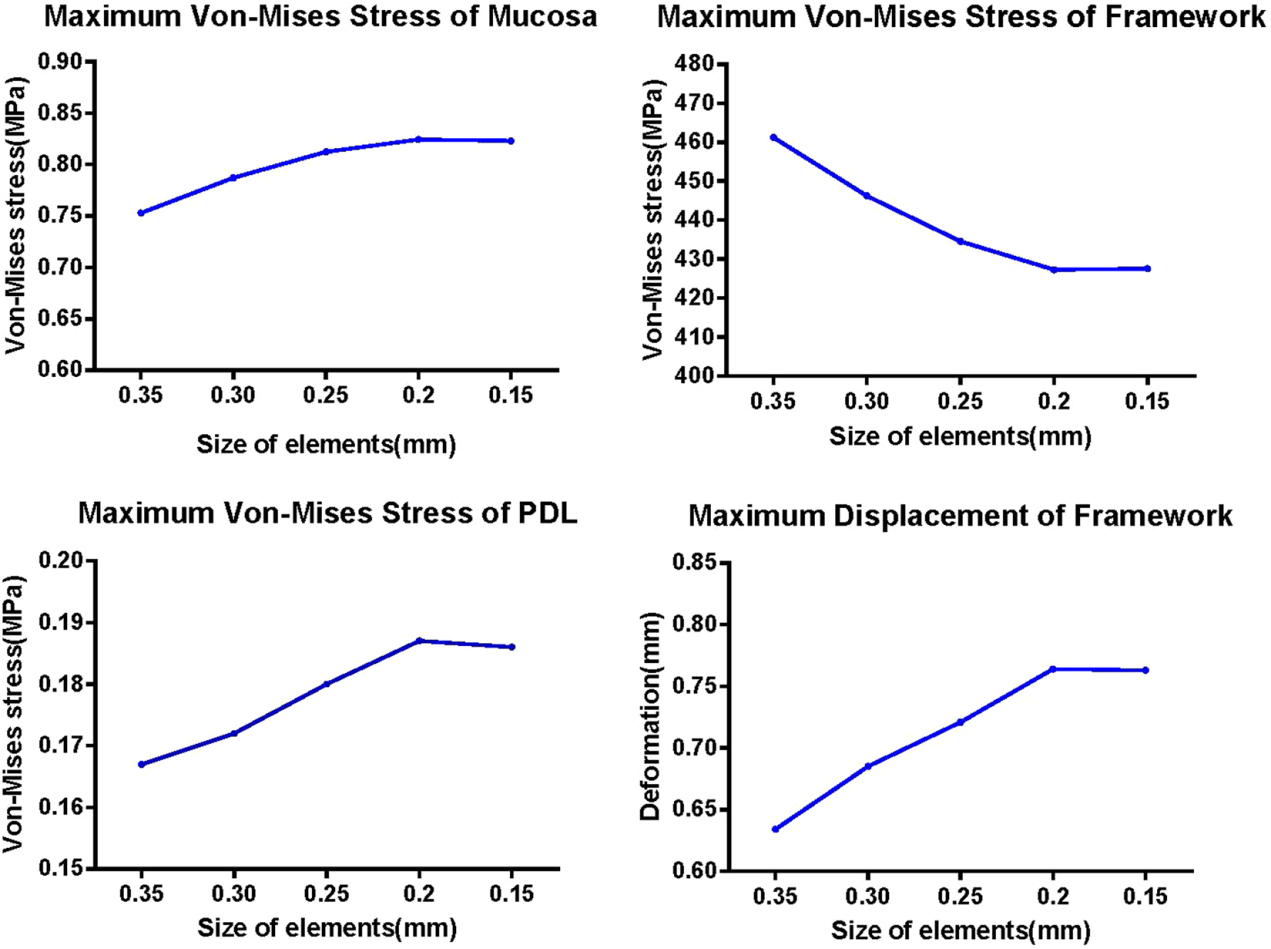
Convergence study: Influence of the size of elements on maximum Von-Mises stress and maximum displacement of finite element model.

The FMBM, FMM, RTMA and all the frameworks, denture bases and denture teeth models were then processed by Abaqus/CAE (2016, SIMULIA Co, USA) to convert into a three-dimensional FEA solid model (Fig. 5). The ten-node tetrahedral elements were selected for the models. A convergence study was carried out to determine the optimal size of elements. In Fig. 6, the influence of the size of elements on the maximum Von-Mises stress and maximum displacement of the model is presented. It shown that the stress and displacement were converged as the element size smaller than 0.2 mm. As a result, the size of elements can be located as 0.2 mm.

All materials except the PDL were assumed to be linearly elastic, homogenous and isotropic to simplify the calculations. Table 1 showed the elastic modulus and the Poisson ratio for each material^43–45^. As for the PDL, the nonlinear hyper-elastic model was used based on the double linear stress-strain curve of Vollmer’s^28^ research: when the dependent variable of PDL ɛ < 7.5%, E1 = 0.05 MPa; and when ɛ > 7.5%, E2 = 0.22 MPa. Also, there are several studies used nonlinear parameters with mucosa, previous studies showed that with RPD scenario, the simulation results with linear mucosa parameter were highly correspondence with *in vitro* test results with sensors^46^, indicating linear parameter of mucosa is acceptable under normal occlusal force with RPD scenario. There are also several studies used the same method to investigate the stress and displacement of mucosa with RPD, which can simplify calculation process as well as obtain accurate results^20,47,48^.

**Table 1:** Material properties of finite element models.

Material	Elastic Modulus (MPa)	Poisson Ratio
Mucosa	3.45	0.45
Denture Base	2,200	0.31
Cancellous Bone	1,370	0.30
Cortical Bone	13,700	0.30
PDL	Non-linear (see below)	0.45
Tooth Dentin	18,600	0.30
Denture Tooth	1,960	0.30
Co-Cr Alloy	235,000	0.33
Titanium Alloy	11 * 10^4^	0.35
PEEK	4,100	0.4

The tooth was simplified as a uniform dentine material without concerning about the difference between the dentine and the enamel, as the mechanical property of these two materials are proved to be similar in the previous study^49^. The PDLs and teeth roots, the denture teeth and denture base were considered as position constraints. The interfaces between the clasps and the remaining teeth were modeled as frictional contacts with appropriate friction coefficients (μ = 0.1), and the friction coefficients between the denture base and mucosa was assumed as μ = 0.01^43,44^.

To simulate an occlusal force, a vertical load of 120 N was applied to the occlusal surface of both the artificial first molar^39^. Although different masticatory activities (e.g. grinding) with various loading patterns may affect the optimization outcome, but the effects of other masticatory activities are less significant compared to the direct biting force because of the magnitudes^50^. The following were investigated: the von Mises stress values of the PDLs, mucosa, frameworks, and the displacement of frameworks. Data were exported to SPSS 19.0 (IBM, Chicago, USA) for statistical analysis. One-way ANOVA and the Student-Newman-Keuls q test was used to determine differences among different framework materials and different framework design schemes. For all comparisons, statistical significance was declared if *p* < 0.05.

## Data Availability

The datasets generated during and/or analyzed during the current study are available from the corresponding author on reasonable request.
